# Key stakeholder views on atrial fibrillation screening: a systematic mixed-studies review and interpretive analysis

**DOI:** 10.1093/europace/euag051

**Published:** 2026-03-14

**Authors:** Kirsty McKenzie, Anushka Jacob, Ben Freedman, Melissa Kilkelly, Rakesh Narendra Modi, Nicole Lowres

**Affiliations:** Department of Heart Rhythm and Stroke Prevention, Heart Research Institute, 7 Eliza St, Newtown, Sydney, NSW 2042, Australia; Faculty of Business, Justice and Behavioural Sciences, Charles Sturt University, Panarama Avenue, Bathurst 2795, Australia; Department of Heart Rhythm and Stroke Prevention, Heart Research Institute, 7 Eliza St, Newtown, Sydney, NSW 2042, Australia; Faculty of Medicine, University of Sydney, Science Rd, Camperdown 2050,Australia; Department of Heart Rhythm and Stroke Prevention, Heart Research Institute, 7 Eliza St, Newtown, Sydney, NSW 2042, Australia; Faculty of Medicine, University of Sydney, Science Rd, Camperdown 2050,Australia; Department of Heart Rhythm and Stroke Prevention, Heart Research Institute, 7 Eliza St, Newtown, Sydney, NSW 2042, Australia; Primary Care Unit, Department of Public Health and Primary Care, Strangeways Research Laboratory, University of Cambridge, 2 Worts’ Causeway, Cambridge, Cambridgeshire CB1 8RN, UK; Department of Heart Rhythm and Stroke Prevention, Heart Research Institute, 7 Eliza St, Newtown, Sydney, NSW 2042, Australia; Faculty of Medicine, University of Sydney, Science Rd, Camperdown 2050,Australia

**Keywords:** Systematic review, Atrial fibrillation, Screening, Opinions, Perspectives, Cardiovascular

## Abstract

**Aims:**

It is essential to understand the key barriers and stakeholder needs related to screening to focus efforts for designing appropriate programmes. Therefore, this study aimed to synthesize the existing literature to understand the pertinent concepts and requirements from key stakeholders regarding implementation of atrial fibrillation (AF) screening.

**Methods and results:**

Database searches were run in MEDLINE via Ovid, Embase via Ovid, CINAHL via Ebsco, PsycInfo via Ebsco, Scopus, and Web of Science Core Collection using specified keywords; supplemented by Google and grey literature searches. Original research papers were included if they contained stakeholder views on implementation of AF screening. A critical interpretive synthesis of data was performed. From 13 332 titles/abstracts, 105 full texts were reviewed and 34 papers included (16 qualitative; 8 surveys; 10 mixed-methods). Significant evidence gaps were identified related to systematic and population-wide screening programmes; and views from system-level stakeholders/key decision-makers. The key themes were: (i) VALUE, BENEFITS AND RISKS OF SCREENING: Stakeholders were cautiously optimistic, liked enhanced practice roles; and positive about health benefits. Concerns raised about potential risks/harms (e.g. anticoagulation), worry for patients, and increased burden for the practice/healthcare system. (ii) PERSPECTIVES ON APPROPRIATE MODELS: Systematic screening not supported by evidence; risk-based approaches suggested; handheld electrocardiogram perceived as quick and easy-to-use; concerns raised over direct-to-consumer devices. (iii) FACTORS IMPACTING IMPLEMENTATION WITHIN HEALTHCARE SETTINGS: Time constraints, impact on workflow, remuneration/reimbursement, and data systems and data security problems were the most common barriers. (iv) SYSTEMIC BARRIERS: These included the need for evidence of benefit; clear guidelines and pathways; adequate remuneration/reimbursement; importance of inter-agency collaboration; software; and access and inclusivity for all patients.

**Conclusion:**

Atrial fibrillation screening is acceptable however definitive evidence regarding need and harms is required. Implementation will require collaboration across healthcare sectors; local solutions; equitable access; remuneration/reimbursement; defined responsibilities and clear pathways; consideration of integration of complex systems; and data security solutions. Given the central importance of system-level barriers, more research is needed on the perspectives and needs of system-level stakeholders, key decision-makers, and consumer groups. Additionally, further research is required to identify strategies for how to address barriers in specific healthcare jurisdictions.

**Registration:**

PROSPERO (CRD42023400110)

## Graphical Abstract

**Graphical Abstract:**
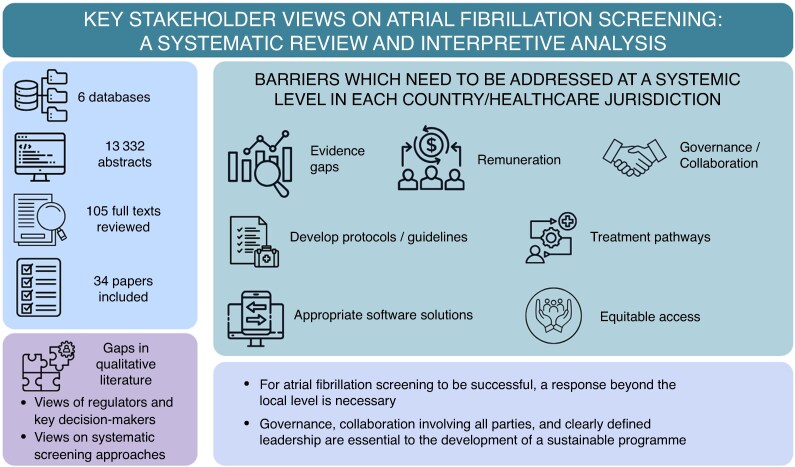

What’s new?

- This study identifies significant gaps in the existing qualitative evidence base related to systematic and population-wide screening programmes; and views from system-level stakeholders and key decision-makers.
- This study synthesizes the key barriers and issues that need to be considered at a systems level prior to implementation, including operational and implementation issues.
- This study identifies the key requirements of each stakeholder group, which need to be met for a screening programme to be considered feasible.
- Our findings show that if AF screening is to be successful, a response beyond the local level is necessary in order to address all barriers and ensure all stakeholder needs are met.
- Governance, collaboration involving all parties, and clearly defined leadership are essential to the development of a sustainable programme.

## Introduction

Detecting atrial fibrillation (AF) earlier has the potential to reduce the incidence of stroke—one of the largest causes of worldwide mortality.^1^ The evidence for systematic screening for AF is inconclusive due to neutral results from the first major randomized control trials (RCTs) of screening which were underpowered.^2–4^ However, a study-level Meta-Analysis of these trials did indicate a significant effect on stroke^5,6^ and the largest definitive screening trial is yet to report.^7^ Opportunistic screening and local pilots are increasingly common in an attempt to stem the morbidity and mortality associated with AF. If forthcoming trials show a net benefit, and it is deemed that all criteria for a National Screening Programme are met,^8,9^ then it is important to understand what is required to implement screening at scale. Whether opportunistic or systematic, local or large scale, the implementation of programmes will be key to maximizing the benefit wrought from screening for AF and minimizing the harms.^10–47^

To design and implement a screening programme at scale, the World Health Organization (WHO) states that it is essential to understand the issues and barriers, and the needs of the key stakeholders.^8–11^ These stakeholders include consumers, healthcare professionals (HCPs), practice staff, and organizations whose support for a programme would be important for success. It is also important to ensure anticoagulation for screen-detected cases when developing a feasible and sustainable solution.^8,12,13^ Multiple AF screening studies have performed qualitative and/or process evaluations evaluating stakeholder needs and perspectives on key issues including benefits and risks of screening, issues pertinent to implementation of specific screening approaches, and broader systemic issues. There has been no synthesis of this evidence. Compiling and synthesizing the breadth of qualitative results will provide robust recommendations and frameworks for AF screening.

Therefore, we performed a systematic review to understand the needs and opinions of the key stakeholders in relation to issues and barriers that underpin the design and delivery of AF screening; map the requirements for different stakeholder groups; underline models that could have potential; and chart significant issues that need to be addressed for the successful implementation of AF screening programmes.

## Methods

### Study design

A systematic review of mixed-methods studies and a critical interpretive analysis was performed in accordance with the preferred reporting items for systematic reviews and Meta-Analysis.^13^ The protocol was registered on PROSPERO (CRD42023400110). Reporting of the results was done according to the framework of the enhancing transparency in reporting the synthesis of qualitative research.^14^

### Key research questions

1. To understand the needs and opinions of key stakeholders in relation to issues and barriers that underpin the design and delivery of AF screening.
2. To explore: (i) the perceived value of screening; (ii) the perceived risks and harms of screening; (iii) appropriate models; (iv) factors impacting implementation of screening within healthcare settings; (v) systemic issues; and (vi) summarize the requirements for acceptability for different stakeholder groups.

### Eligibility criteria

Studies were required to be peer reviewed and ethically approved. Studies were included if they met all three inclusion criteria: (i) any stakeholder relevant to screening for AF, including its implementations. Stakeholders may include members of the public, patients, healthcare professionals, practitioner groups, community support groups, policy makers, commercial providers, and academics. Stakeholders may come from any setting (e.g. community, general practice, pharmacy, hospital, government); (ii) studies using qualitative methods, surveys, and process evaluations; including interviews, focus groups, observations, evaluations, documentary and media analyses, and other related approaches; and (iii) primary studies that report stakeholder views and attitudes towards screening for AF and its implementation. Studies were excluded if they met any of the following exclusion criteria: (i) AF detected by implanted devices; (ii) quantitative studies that did not report views or opinions in the results; and (iii) review papers or viewpoint articles.

### Search strategy

The search was designed and tested by a health librarian (Ms. Isla Kuhn) in collaboration with Dr. Jenni Burt (senior social scientist). The search was based on the Population, Exposure, and Outcome Framework.^15^ Database searches were run in MEDLINE via Ovid, Embase via Ovid, CINAHL via Ebsco, PsycInfo via Ebsco, Scopus, and Web of Science Core Collection in February 2023 and repeated in May 2025. The search was limited to English language papers, published from 2005 onwards to ensure relevant contemporary data. The keyword search strategy, search terms, and controlled vocabulary such as MeSH terms (in MEDLINE) and EMTREE (in Embase) are provided in Supplementary material online, *Appendix S1*. We also conducted Google and targeted searches for relevant grey literature, consulting selected institutional websites related to screening delivery.

Records identified by the database search were imported into Rayyan. Duplicates were removed. Two authors (N.L. and A.J.) independently performed the title and abstract screening, identified relevant studies, and independently reviewed full texts against the eligibility criteria. Full texts were exported into Endnote. A search of the reference lists for all identified studies was performed, using both forward and backward citation searching. At each stage, consultation occurred with all authors to resolve differences and obtain consensus on included articles.

### Data extraction

A data extraction template was developed using Excel and pilot tested to ensure appropriate information was extracted. Data extraction included: author, year of study, funding, country data were collected, study aim, nature of AF screening approach explored (e.g. opportunistic, systematic), study design, data collection method, setting, sample size, stakeholders, and key findings. Data extraction was conducted by three reviewers (M.K., K.M., and N.L.) independently and discussed with all authors to gain consensus.

### Quality assessment

To assess risk of bias, two independent reviewers (N.L. and M.K.) used the Mixed Methods Appraisal Tool (MMAT, version 2018).^16^ The MMAT was chosen as it critically appraises quantitative, qualitative, and mixed-methods studies, as were included in this review.

### Data synthesis

A critical interpretive synthesis of data was performed independently by two researchers (K.M. and M.K.), and the evidence was synthesized with critical interpretation. Key findings across included studies were extracted from results of the included papers and coded to identify recurring issues and barriers. An inductive approach was used to create a series of core themes, with consideration given to how themes related to our research questions. Rigour was ensured by engaging reflexively as a team; iterative analysis and close consideration of the identification and evidencing of themes and having two researchers read and undertake coding independently.

## Results

**Figure 1:**
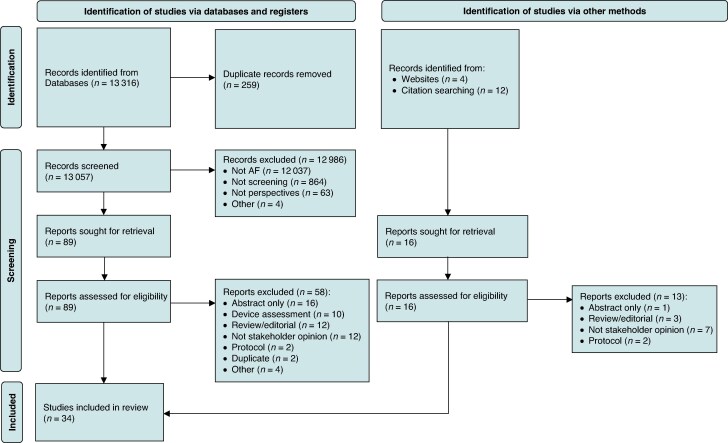
PRISMA flow chart. AF, atrial fibrillation.

The initial search identified 13 332 records, and 105 full texts were retrieved and assessed for eligibility, as outlined in the PRISMA flow chart (*Figure 1*). Thirty-four original research articles were included in the review,^17–50^ with varied study designs: 16 qualitative, 8 quantitative descriptive (i.e. surveys), and 10 mixed-methods studies (*Table 1*; see Supplementary material online, *Appendix S2*). All but two studies^19,46^ scored highly on the MMAT quality assessment (see Supplementary material online, *Appendix S3*), however these studies were not removed as results aligned with overall results. Details and broad findings from each paper are summarized in Supplementary material online, *Appendix S2*.

**Table 1:** Study characteristics

Study	Country	Screening approach/setting	Methodology	Stakeholders
A'Court *et al*.^17^	United Kingdom	Consumer-led wearables	Mixed methods: 6 semi-structured interviews 3 focus-group discussions Content analysis of clinic letters	20 cardiology healthcare professionals GP (*n* & 10) Cardiologist (*n* & 6) Cardiology Trainees (*n* & 2) Nurse Practitioners (*n* & 2)
Atlas *et al*.^18^	United States of America	Opportunistic single screen General practice Handheld ECG Age 65+ years	Quantitative: Survey	153 primary care physicians
Bleske *et al*.^19^	Mexico	Opportunistic single screen Community pharmacy Handheld ECG Age 50+ years with risk factors	Mixed methods: 10 surveys 5 interviews	10 patients 5 pharmacists
Boriani *et al*.^20^	Europe, Asia/Oceania, Americas	Consumer-led wearables (hypothetical)	Quantitative: Survey	588 cardiology healthcare professionals Recruited from AF-SCREEN International Collaboration
Callanan *et al*.^21^	Ireland	Opportunistic single screen (hypothetical) General practice	Qualitative: 10 structured interviews	8 general practitioners
da Costa *et al*.^22^	Europe, United Kingdom, Asia/Oceania, Canada	Opportunistic single screen Pharmacy awareness campaign Pulse palpation Age 40+ years	Mixed methods: Outcome data from screening 10 semi-structured interviews	10 pharmacists (county co-ordinator of Awareness campaign)
Ding *et al*.^23^	Americas, Asia Pacific	Consumer-led wearables (hypothetical)	Quantitative: Survey	1601 cardiac healthcare professionals Recruited from Heart Rhythm Societies Electrophysiologist (*n* = 694) Cardiologists (*n* = 151) Fellows (*n* = 204) Other physician (*n* = 50) Advance practice practitioner (*n* = 185) Nurse (*n* = 121) Other, e.g. trainee (*n* = 188)
Ding *et al*. ^24^	United States of America	Systematic prolonged screening Stroke survivors Smartwatch PPG for 14 days Age 50+ years	Mixed methods: 90 System Usability Scale surveys 10 in-depth interviews	90 stroke survivors (age 50 + years)
Engler *et al*.^25^	Europe (11 countries)	Four different approaches (hypothetical) Opportunistic single time point Opportunistic prolonged Systematic single time point/prolonged Population-wide patient-led	Qualitative: 24 semi-structured interviews	13 cardiac healthcare professionals (cardiologists/general practitioners) 11 regulators
Hall *et al*. ^26^	United Kingdom	Opportunistic single screen Diabetes Clinic Handheld ECG Diabetes patients	Qualitative: 9 semi-structured interviews	9 patients with diabetes
Hamilton *et al*. ^27^	United Kingdom	Systematic prolonged screening Primary care sites Handheld ECG recordings for 3 weeks High-risk patients Age 30+ years	Qualitative: 23 semi-structured interviews Nested within the Future Innovations in Novel Detection of AF (FIND-AF) study	15 healthcare professionals General practitioner (7) Nurse (4) Physician associate (1) Administrative staff (2) Practice manager (1)
Hassan *et al*.^28^	United Kingdom	Opportunistic single screen Dental practice Handheld ECG	Qualitative: 11 semi-structured interviews	11 dental practice staff (dentists, dental therapists, dental nurses and managers)
Hoare *et al*.^29^	United Kingdom	Systematic prolonged screening General practice Handheld ECG self-recordings daily for 4 weeks Age 65+ years	Qualitative: 23 semi-structured interviews Nested within the SAFER study	23 patients (age 65+ years)
Hoare *et al*.^30^	United Kingdom	Systematic prolonged screening General practice Handheld ECG self-recordings daily for 4 wks Age 65+ years	Qualitative: 50 semi-structured interviews Nested within the SAFER study	50 decliners of AF screening
Koshy *et al*.^31^	Australia	Opportunistic prolonged screening Hospital (emergency, coronary care, and intensive care units) smart technology (watches and handheld ECG) Cardiac patients Age 18+ years	Quantitative: Survey	363 patients (age 55–80 years)
Lown *et al*.^32^	United Kingdom	Opportunistic single screen General Practice Nurse-led Multiple screening devices (blood pressure meter, handheld ECG, and two ECG wearables) Age 65+ years	Qualitative: 15 semi-structured interviews Nested within the Screening for Atrial Fibrillation using Economical and Accurate Technology (SAFETY) study	15 patients
Lowres *et al*.^33^	Australia	Opportunistic single screen Pharmacy Handheld ECG Age 65+ years	Qualitative: 9 semi-structured interviews Nested within the SEARCH-AF study	9 pharmacists
Macniven *et al*.^34^	Australia	Opportunistic single screen Aboriginal Controlled Community Health Services Handheld ECG Age 45+ years	Qualitative: 18 semi-structured interviews (including quantitative and qualitative questions) Nested within a screening study	18 staff (Aboriginal health workers and registered nurses)
Manninger *et al*.^35^	Europe (42 countries)	Consumer-led wearables (hypothetical)	Quantitative: Survey	417 physicians (cardiologists and electrophysiologists; median age 37 years, IQR 32–43) Recruited from European Heart Rhythm Association Young EP, members of national EP working groups and via social media platforms
Manninger *et al*.^36^	Europe (51 countries)	Consumer-led wearables (hypothetical)	Quantitative: Survey	539 physicians (cardiologists and electrophysiologists; median age 38 years, IQR 34–46) Recruited from European Heart Rhythm Association
McKenzie *et al*.^37^	Australia	Opportunistic single screen General practice waiting rooms Self-screening kiosk Age 65+ years	Mixed methods: 20 semi-structured interviews with practice staff Observations of 22 patients performing screening	22 patients and 20 general practice staff General practitioners (*n* = 6) Receptionists (*n* = 9) Practice managers (*n* = 5)
McKenzie *et al*.^38^	Australia	Screening as a concept (hypothetical)	Qualitative: 25 semi-structured interviews with key stakeholders relevant to AF screening	25 stakeholders representing: Health professionals (*n* = 9) Professional bodies (*n* = 4) Government (*n* = 4) Charities/support groups (*n* = 5) Research (*n* = 8) Industry (*n* = 2)
Orchard *et al*.^39^	Australia	Opportunistic single screen General practice Nurse and reception led screening Handheld ECG Age 65+ years	Qualitative: 14 semi-structured interviews	8 patients and 6 general practice staff General practitioners (*n* = 3) Receptionists (*n* = 2) Nurse (*n* = 1)
Orchard *et al*.^40^	Australia	Opportunistic single screen General practice Flu vaccination clinic Nurse-led screening Handheld ECG Age 65+ years	Mixed methods: Outcome data from screening 17 semi-structured interviews	17 general practice staff *General practitioners (n* = *5*) *Nurses (n* = *7*) *Practice Managers (n* = *5)*
Orchard *et al*.^41^	Australia	Opportunistic single screen General practice GP and Nurse-led screening Handheld ECG Age 65+ years	Mixed methods: 43 semi-structured interviews with practice staff Observation of practice staff Quantitative screening data Nested within the AF-SMART study	43 general practice staff General practitioners (*n* = 21) Nurses (*n* = 13) Practice managers (*n* = 11)
Sabater-Hernández *et al*.^42^	Australia	Opportunistic single screen (hypothetical) Pharmacy Self-monitoring with a rented device Consult with pharmacy and follow up with general practitioner	Qualitative: 4 interviews and 1 focus group with potential service users 1 focus group mixed stakeholders 1 focus group with community pharmacists	8 potential service users (age 65+ with hypertension or AF) 8 mixed stakeholders [service users (*n* = 1) community pharmacists (*n* = 2) general practitioner (*n* = 1) cardiologist (*n* = 1) heart failure nurse (*n* = 1) AF research nurse (*n* = 1) stroke Foundation (*n* = 1)] 4 community pharmacists (owners and managers)
Savickas *et al*.^43^	United Kingdom	Opportunistic single screen General practice Pharmacist-led screening During flu vaccination season Pulse palpation or Handheld ECG Age 65+ years	Qualitative: 6 homogeneous stakeholder focus groups Nested in the Pharmacists Detecting Atrial Fibrillation (PDAF) study	25 patients 4 clinical pharmacists 9 general practice staff
Shih *et al*.^44^	United States of America	Consumer-led wearables	Qualitative: 19 semi-structured interviews	19 Apple Watch consumers
Taggar *et al*.^45^	United Kingdom	Opportunistic single screen (hypothetical) General practice	Mixed methods: survey using Likert scale questions and free-text open-ended questions	212 healthcare professionals General practitioners (*n* = 118) Practice nurses (*n* = 50) Nurse practitioners (*n* = 17) Healthcare assistants (*n* = 27)
Theunissen *et al*.^46^	Netherlands	Opportunistic single screen General practice Nurse-led screening From diabetes management OR cardiovascular risk management programmes Handheld ECG Age 65+ years	Mixed methods Survey 15 semi-structured in-depth interviews Nested within a screening study.	74 practice nurses
Uittenbogaart *et al*.^47^	Netherlands	Opportunistic single screen General practice General practitioner, nurse, or healthcare assistant led screening Pulse palpation; blood pressure monitor; and handheld ECG Age 65+ years	Qualitative: 7 ‘group’ semi-structured interviews with a mix of healthcare professionals Nested within The Detecting and Diagnosing Atrial Fibrillation (D2AF) study	15 general practice healthcare professionals (general practitioners, nurse practitioners, and healthcare assistants)
Vermunicht *et al*.^48^	Europe (18 countries)	Opportunistic single screen (hypothetical) General practice Handheld ECG	Quantitative: Survey	659 general practice professionals General practitioners (*n* = 620) Nurses (*n* = 20) Other (*n* = 19)
Wong *et al*.^49^	Australia	Opportunistic single screen (hypothetical) General practice	Quantitative: Survey	463 general practitioners
Wong *et al*.^50^	Australia	Community-advertised prolonged screening Handheld ECG recordings for 1 year Age 75+ years	Qualitative: 7 ‘group’ semi-structured interviews with a mix of healthcare professionals Nested within The Mass Atrial Fibrillation Screening study	48 Patients 11 general practitioners

AF, atrial fibrillation; ECG, electrocardiogram; GP, general practitioner; IQR, inter-quartile range.

The majority of studies (24/34) assessed views on opportunistic screening: 17 evaluating a specific intervention^18,19,22,26,28,31–34,37,39–41,43,46,47,50^ and 7 generalized views.^21,25,38,42,45,48,49^ Only six studies explored perspectives on systematic screening: two from the SAFER study,^29,30^ one from FIND AF study,^27^ one post-stroke study^24^, and two generalized views.^25,38^ Consumer-led screening was assessed in six studies: two assessing an intervention^17,44^ and four generalized views.^20,23,35,36^ Only two studies assessed the views of public health organizations (i.e. views of stakeholders other than consumers, HCPs, or healthcare staff).^25,38^

The included studies comprised a range of stakeholders (*Table 1*; see Supplementary material online, *Appendix S2*). In this review, the stakeholders have been grouped into (i) consumers; (ii) HCPs [general practitioners (GPs), cardiologists, nurses, aboriginal health workers, dentists, pharmacists]; (iii) practice staff (non-HCP staff including receptionists and practice managers); and (iv) public health organizations (charities/advocacy groups, research, government and regulators, and industry). The perspectives of these four stakeholder groups, in relation to the following areas, are reported in detail below:

- Value, benefits, and risks of screening;
- Perspectives on appropriate models;
- Factors impacting implementation of screening within healthcare settings;
- Systemic issues.

As shown in *Table 2*, there were broad areas of agreement between stakeholder groups on key aspects of screening including perceived value of screening for stroke reduction; acceptability of hand-held devices in GP settings; barriers in healthcare settings related to time constraints, impact on workflow and lack of remuneration; need for evidence of benefit; value of risk-based approach; and need to address access and equity in healthcare systems.

**Table 2:** Stakeholder perspectives summary

Perspective	Consumers	HCPs	Reception staff, practice managers	Public health organizations, researchers, private companies, regulators
Value of screening	Like opportunity to be tested for something that would otherwise not be detected, and potentially prevent stroke Some do not want to be screened	Stroke prevention Improved cardiovascular profile of practice	Stroke prevention	Stroke prevention Reduced burden on healthcare system
Risks/harms	Worry, cost, inconvenience	Patient worry, bleed from coagulation, unnecessary referrals, impact on workflow	Impact on workflow	Patient worry, risk of bleed from anticoagulation, overload healthcare system
Appropriate models	Like the handheld ECG more than traditional 12-lead ECG Prefer GP setting to community pharmacy May not trust direct-to-consumer devices, but may still seek advice after positive result	Handheld single lead ECG is convenient, portable, and easy to use, more accurate than pulse palpation and less time consuming than 12-lead ECG GP-led screening using a handheld device; identification of high-risk patients; integration with other health programmes or into standard care Pharmacy-led screening; identification of high-risk patients Direct-to-patient devices have potential but want more evidence and guidance on their use and clinical utility; prefer ECG to PPG	Confident to use the handheld ECG; staff led or assisted screening creates time burden	GP-led screening; identification of high-risk patients Incorporate direct-to-consumer devices
Barriers and facilitators in healthcare settings		Time constraints and workflow in healthcare settings; GPs prefer other staff to screen; patients may not be screened in busy periods Lack of equipment Lack of remuneration/reimbursement A practice champion	Time constraints and workflow in healthcare settings; patients may not be screened in busy periods	Time constraints and workflow in healthcare settings; screening everyone is not tenable
Systemic issues		Need for evidence Importance of guidelines, protocols and pathways—value in identification and screening of higher risk individuals Data integration and transfer Inequalities in healthcare system Remuneration/reimbursement		Need for evidence Value in identification and screening of higher risk individuals Need for inter-agency collaboration Inequalities in healthcare system Remuneration/reimbursement

ECG, electrocardiogram; GP, general practitioner; HCPs, healthcare professionals; PPG, photoplethysmography.

### Value, benefits, and risks of screening

There was widespread support for AF screening across all stakeholder groups (*Table 2*). Staff working in primary practice and other healthcare settings saw the value of screening, both as a general good, and in some cases within their own practice. Benefits included the opportunity to increase detection rates, and prevent stroke; improved cardiovascular profile in the practice; and enhanced responsibility for health, and reassurance for patients.^21,27,37,38,40,41,50^ In addition, pharmacists and dental nurses valued the opportunity to increase skills and take on new responsibilities, and the opportunity to save lives.^28,33,43^ Researchers and those working in public health identified the importance to reducing the burden of disability and death associated with stroke, both for individuals, and the wider community.^38^ While most stakeholders were broadly positive, some expressed concerns about potential risks/harms, including creating burden and worry for patients, risks associated with anti-coagulation; and increased burden for the practice and for the healthcare system.^21,27,37,38^

Screening was also well received by consumers. Consumers across studies knew very little about AF, but valued the opportunity to take part in screening, either in primary care or community settings, or at home.^26,29,37,40,50^ Across studies, consumers valued AF screening because they perceived that early detection could help prevent stroke and provide peace of mind. Consumers’ concerns included being overserviced, cost, inconvenience, and anxiety.^26,29,50^ Not all consumers took up the opportunity to take part in specific screening programmes (embedded in research programmes).^30,37^ Reasons included not believing that screening was important, not wanting to know if there was something wrong, and inconvenience.^30^

### Stakeholder perspectives on appropriate screening models

**Figure 2:**
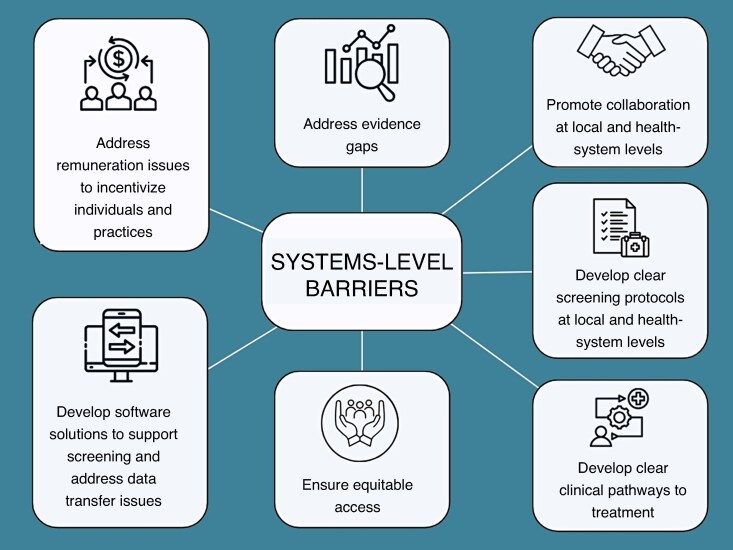
Barriers to overcome at a systemic level in each country/healthcare jurisdiction.

Only two papers considered the perspectives of a wide range of stakeholders regarding different models for screening, including systematic and opportunistic approaches.^25,38^ Most papers assessed stakeholder perspectives on a specific intervention, most commonly the use of handheld single-lead electrocardiograms (ECGs) within a healthcare setting (general practice, pharmacy, dentistry). The literature identified some models that have broad stakeholder acceptability, although as noted in the following sections there is a need to first address local and systemic barriers (*Figure 2*) to ensure the successful integration of these models.

#### Systematic screening

Although only assessed in two papers,^28,41^ systematic screening of all in-scope individuals (e.g. through a mailout to all people over 65, or within primary care via an invite) was seen as being untenable and not supported by the limited current evidence.^25,38^ However, there was some support for systematic screening using a risk-based approach, focused on identification and screening of individuals at higher risk of having AF who had additional risk factors for stroke.^18,27,38,43,48,50^ Suggested approaches included systematic identification of patients using a set of risk factors and inviting these in for screening, or screening all at-risk patient groups in clinics (e.g. diabetes).^26,38^

#### Opportunistic screening using a handheld electrocardiogram

Several papers identified that opportunistic GP-based screening (using a single-lead ECG) was seen as an appropriate model for screening.^25,38,47,48^ Other locations where handheld devices could be incorporated included pharmacy and dental.^19,28,33,43^ The handheld ECG was widely acceptable across all stakeholder groups. Across studies, HCPs perceived the handheld ECG to be quick and easy to use, with GPs, reception staff, nurses, pharmacists, dentists, and Aboriginal health workers all expressing confidence to use such a device.^28,33,34,39–41,46,47^ However, one study found HCPs initially preferred to ‘confirm’ the handheld single-lead ECG results using pulse palpation, but as they became more familiar with the device their confidence in device accuracy increased.^36,40,42,46,47^

#### Direct-to-consumer devices

Another potential model is the incorporation of direct-to-consumer devices with AF detection capabilities, utilizing either ECG (e.g. Kardia-pro; some Apple watches) or photoplethysmography (PPG) (e.g. some Apple Watches, Fitbit). These were seen as having potential or even as representing a great opportunity.^20,38^ However, many stakeholders have significant reservations including concerns about consequences of false positives (e.g. patient anxiety, unnecessary referrals, and risk of bleeding if anticoagulant medication is prescribed), as well as data transfer and security, and increased burden on the healthcare system.^17,25,38^ There was a perception that patient-led screening using direct-to-consumer devices could increase workload as patients would come in to discuss results with a GP, which could ‘overload the system’.^17,38^

Despite these concerns, stakeholders also perceive that direct-to-consumer devices for AF screening are inevitable,^17,20,25^ and several studies found that some HCPs are recommending them to patients to screen for AF,^20,23^ and using data provided by patients in clinical decision-making.^17^ Healthcare professionals may have more confidence in ECG-based devices over PPG-based devices, being more likely to recommend such devices, as well as diagnose AF, order follow-up diagnostic tests, or initiate anti-coagulation therapy.^35,36^ Healthcare professionals report concerns about the clinical utility of data on AF from direct-to-consumer devices and would like to see more research on the accuracy of direct-to-consumer devices.^17^

#### Consumer perspectives

Consumers who were screened in HCP settings using a handheld device were appreciative of the opportunity to be screened, engaged with the technology, and found it more convenient than a 12-lead ECG.^33,37,39,43^ Consumers were also happy to engage in self-screening using direct-to-consumer devices such as smartwatches with ECG. Consumers value their convenience, comfort, appearance, and safety of these new devices and prefer them to traditional approaches such as 12-lead ECG and Holter monitors.^24,29,31^ Consumers also appreciated the non-invasive nature of handheld or wearable devices for extended monitoring.^24,29,32,50^ Recommendation by an HCP was associated with trust and engagement in extended home-based screening,^32,50^ while consumer refusal was associated with inconvenience, and concerns about compromising the research project.^30^ There is some evidence that consumers may lack trust in direct-to-consumer wearables, as they believe that they are not as accurate as a physician interpreted test.^31,44^ However, one study found 92% of consumers would follow up a diagnosis from a direct-to-consumer device with an HCP, and 91.7% believed them more convenient than a 12-lead ECG.^31,34^

### Factors impacting implementation within healthcare settings

Operational barriers impacting implementation included time constraints and impact on workflow, lack of integration between data systems, and remuneration/reimbursement (discussed in the systemic barriers section). Other barriers included lack of equipment, lack of staff, and need for an appropriate space for screening.^19,21,45,48^

#### Time constraints and workflow

Time constraints and impact on workflow were identified as significant issues by staff across settings^37,38,45^ (*Table 2*). Regardless of the screening method used, GPs had concerns about the time taken to undertake screening, and the time taken to review screening results, and impact on workflow if AF was identified.^21,27,39–41,47,50^ However, some GPs did not perceive that there was significant increase in workload^37^ or suggested that the benefits of reduced stroke outweigh the increase in workload.^27^ Approaches where screening was undertaken by other staff in the practice, or by patients themselves, were seen as preferable by GPs,^21,37^ and GPs would prefer to delegate screening to other staff in the practice which could require additional resources.^47^ Reception staff, while confident to use screening devices, were not enthusiastic about being involved in screening due to significant impacts on time, and belief that it was not part of their role.^37,39^ Dental and pharmacy staff also expressed concerns about time and impact on workflow.^28,33^ Across all healthcare settings, staff prioritize normal duties over screening.^37,50^ Screening by GPs, nursing staff, pharmacists, or reception staff may not be possible during very busy times, meaning that some eligible patients will not be screened.^37,50^

#### Data management systems

The need for efficient systems for data management was essential for acceptability.^38,41,43,46^ In many studies, interoperability between systems was identified as a significant barrier.^38,43,46^ Systems that require manual integration of screening data into patient files are cumbersome and add to burden experienced by staff.^38^ There were also concerns about how to integrate data from patient devices into patient records.^23,36^

### Systemic barriers

Some key systemic issues were identified (*Figure 2*) that cannot be addressed at the local level and will require interagency collaboration and government intervention. These include issues with research gaps/lack of evidence, and operational barriers. The identified barriers are discussed below.^28,41^

#### Need for evidence

The need for evidence was strongly expressed by a range of non-consumer stakeholders. This included evidence for the net benefit of AF screening; benefit of systematic screening of asymptomatic individuals; treatment of screen-detected AF; and a cost benefit analysis.^25,38,48^ There was also a perceived need for evidence supporting the clinical utility of direct-to-consumer devices.^20,23,35^

#### Guidelines and protocols

Effective guidelines, protocols, and pathways were seen as important to support screening in healthcare settings. Stakeholders indicated the need for clear protocols and referral pathways for positive results in GP-led screening^21^ and nurse-led screening,^41^ for data transfer and referrals for AF detected in pharmacy or in community settings,^22,38,42^ and protocols for follow-up of high-risk individuals.^27^ Knowledge of guidelines is associated with increased screening by HCPs,^49^ however guideline complexity was identified as a barrier.^21^ Time constraints may also prevent GPs from screening as per guidelines.^38^ Guidelines, protocols, and pathways were also identified as important for consumer-initiated screening using direct-to-consumer devices.^20,23,35^ While HCPs believe that consumer-initiated screening has great potential, they also desire guidelines and advice (e.g. from scientific societies) on the use of such devices for detection of AF, mechanisms for managing positive results, and regulation by authorities.^20,23,35^

#### Collaboration and communication

Collaboration between agencies is required to facilitate a system-wide response to AF screening.^38^ In addition, several studies identified a need for effective collaboration and formalized communication methods between pharmacy and general practice to ensure that pharmacy-based programmes are effective.^22,42^ Pharmacists anticipated low levels of success due to difficulties in communication with GPs arising from misunderstandings associated with professional boundaries.^42^

#### Remuneration and reimbursement

Stakeholders across different settings (general practice, pharmacy and dental practice) reported that remuneration/reimbursement is required if screening is to be effective and sustainable.^28,38,42,45,50^ Effective remuneration/reimbursement was seen to facilitate implementation and incentivize screening.^33,40^ Several studies identified improvements to remuneration/reimbursement as essential for implementation of a screening programme.^25,38^ Local healthcare systems are not set up to remunerate GPs or others to undertake screening.^25,38^ However, integrating remuneration/reimbursement into existing frameworks could be complex, such as calculating remuneration/reimbursement in dental practice.^28,41,45^

#### Access and equity

Screening is dependent on access to healthcare services, however consumers may not have access to healthcare due to lack of services in the area, or capacity to pay for such services.^21,25,38^ Cost of attending appointments, and cost of digital devices, was identified as a barrier for consumers to engage in screening.^17,21,26,31,32^ In addition, several studies indicate that digital literacy, especially for older patients, may be an issue for self-screening.^17,30,31,37^ Many patients required reception staff assistance to complete self-screening using a self-service check-in in GP waiting rooms.^37^ Digital literacy and device complexity were also barriers for patient-led screening at home, especially for older people.^17,31^ Furthermore, some patients declined to participate in a screening programme because they were concerned about the technical aspects of screening using an at-home device.^30^

Ensuring that all consumers can access screening, regardless of their income, location, or capacity to engage with technology, was identified as an important consideration.^25,38^ However, there was very little discussion of how to achieve this goal in specific contexts. Some suggestions, which may increase access for patients with lower digital literacy, were simpler interfaces^24,37^ and patient training for using the self-screening device.^24,28,41^

### Implementation strategies for addressing barriers

A set of general recommendations to address key barriers to facilitate implementation were included in two papers,^25,38^ and these are incorporated in *Figure 2*. These recommendations are general in nature and do not provide concrete strategies for implementation in local jurisdictions.

Some papers suggested specific strategies to address barriers, however these initiatives are mostly specific to the relevant healthcare system and country. Implementing these strategies may be beyond the scope of individual practices, and would require funding, investment in software and equipment, additional staff or staff training.

To address time constraints and impact on GP workload, the following strategies were identified:

- incorporate screening within other existing healthcare programmes, e.g. flu vaccines, national health screen, healthy heart check (Europe, UK, Australia^27,30,38,40,41,48,51^);
- incorporate screening into the general practice check-in process (Australia^37,38^);
- extra staff and/or train non-GP staff to perform screen (Ireland, Europe, Australia^21,25,40^);
- utilize integrated software in primary care to identify patients most in need of screening (UK, Europe, Australia^27,41,48^);
- use software pop-ups to remind GPs to screen (UK, Australia^27,41,48^);
- invest in equipment^24,28,43^ (e.g. handheld devices) (Europe, Australia^25,41^).

Other strategies focused on overcoming remuneration issues. Except for user-pay models for rental of a device, remuneration/reimbursement models require a solution above the level of the individual practice. Suggested remuneration/reimbursement models in general practice included:

- incorporation of AF screening into reimbursement frameworks so that GPs can bill for screening (e.g. Medicare item numbers in Australia), or incentive payments for the practice (Australia^38^);
- remuneration/reimbursement in pharmacy could include a nominal patient fee for renting a screening device or government incentive payments to the pharmacy (Australia^42^).

## Discussion

**Figure 3:**
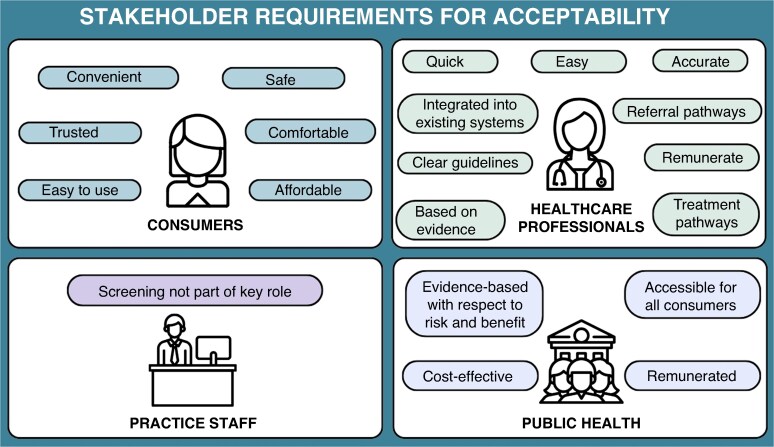
Stakeholder requirements for acceptability.

To our knowledge, this is the first study to systematically synthesize the views of a range of stakeholders on AF screening. Our review identified significant gaps in the existing body of literature. There is a lack of qualitative research on systematic and population-wide screening programmes^2,3^; only two papers assessed system-level stakeholders/key decision-makers (e.g. government, policy makers, funders, advocacy groups)^25,38^; and most of the research is focused on acceptability of a specific intervention or device, rather than screening as a whole. Our review identified a set of barriers which need to be addressed at a systems level when designing a suitable screening programme (*Figure 2*). Our review also identified a set of stakeholder requirements that need to be met for screening to be acceptable and feasible (*Figure 3*). Our synthesis can be utilized to provide guidance on implementation of screening in local jurisdictions. When considering an implementation strategy, decision-makers need to consider whether the programme meets the identified needs of stakeholders (*Figure 3*), and how the systemic issues (*Figure 2*) can be addressed within the local jurisdiction.

The best approaches will facilitate screening that is easy and quick; that is supported by evidence and resources, and by appropriate protocols and pathways; and that is accessible for all consumers. The findings identified some models that have stakeholder acceptability, such as opportunistic screening with a handheld ECG in general practice. However, there is no single screening model that currently addresses all needs. For example, screening with handheld ECG in general practice has consumer and HCP stakeholder acceptability (easy, comfortable, convenient, quick, accurate), but there are many barriers still to overcome (time and workflow burdens, currently not remunerated/reimbursed, and may not be affordable or accessible for all patients). Access is a critical issue. When choosing a model, decision-makers will need to identify whether this model will reach and be accessible to the intended target population. Appropriate solutions in any given health jurisdiction may require multi-faceted approaches that do not rely solely on screening of patients in primary care.

Barriers were identified at both the local level and the^7–9,52^ systemic level. Addressing these will necessitate a response beyond the local level, involving all parties. Our findings state that governance and collaboration, and clearly defined leadership are essential if AF screening is to be successful. These findings are consistent with current best practice for development of screening programmes which, since Wilson and Jungner criteria in 1968,^9^ have evolved to include more focus on systems issues.^8,51^ Best practice now includes consideration of operational and implementation issues, infrastructure requirements, acceptability of entire programmes rather than the individual test, and programme management systems.^8,51^ The WHO states that clearly defined leadership, co-ordination, management, and accountability are essential to the success of any screening programme.^8^ The WHO also outline the requirement for clear steps in the screening pathway that include treatment/follow-up, reporting of outcomes, and evaluation of the screening programme.^11^ In addition, screening programmes require good information systems, careful oversight, and quality assurance evaluation.^8^

The identified need for evidence to justify screening is aligned with WHO screening criteria which outline the need for evidence of the balance of benefits and harms, and cost effectiveness.^8,9^ The need for AF screening RCT evidence is well known and highlighted in this review. Large-scale RCTs are currently underway, including the SAFER study and associated cost-effectiveness analysis.^7^ In addition, an individual patient data Meta-Analysis of RCTs in 2022 showed reduction in stroke with AF screening (relative risk 0.91; 95% confidence interval 0.84–0.99).^6^ These trials will be critical in determining appropriateness of AF screening. It is also important that qualitative evaluations of RCTs are performed and disseminated, especially those related to systematic screening studies. There are notable absences in the literature as some major randomized trials on systematic screening (e.g. STROKESTOP,^3^ GUARD-AF,^2^ and VITAL-AF^4^) have not published qualitative evaluations of stakeholder views.^2–4^ This qualitative evidence is essential to address the gap in the current evidence base.

Funding, equity, and access were identified as critical system-level issues for implementation. Appropriate financing mechanisms are vital to success of screening, as remuneration/reimbursement is required to influence HCP behaviour.^11^ However, funding needs to cover all the components of screening including equipment, training, health promotion, testing, treatment, and monitoring.^8^ The mechanism for who pays for screening, and whether this can be equitable for both patients and those providing screening, will look different in different contexts, as funding programmes and access to healthcare differ between countries and health systems. It is beyond the scope of this review to make recommendations on how this issue can be addressed. However, a key implication of our synthesis is that each jurisdiction will need to ensure that the question of funding and access are adequately addressed rather than leaving this issue to local entities.

System-level issues related to e-health devices and consumer-led wearables were identified, which align with expert consensus.^52,53^ These include data security, data transfer, digital literacy, and clinical guidance. Growth in consumer wearables is seen as inevitable, especially in a landscape of proliferating direct-marketed devices for screening, and it is important to be prepared by addressing the known issues early.^17,52,53^ These issues are best addressed at a systems level with good quality governance supported by research, with collaboration and input from government and other stakeholder organizations.

### Strengths and weaknesses

This is the first study to combine views of a range of stakeholders on value and implementation issues for AF screening. There are several limitations to the included studies that warrant consideration and may impact the generalizability of the results and the applicability across different healthcare systems and sociocultural contexts. The included studies are highly heterogenous, and the potential for publication bias should be considered. Most studies did not ask about AF screening as a general concept. There is also a paucity of qualitative literature regarding views on systematic screening. Views in many studies are constrained by the study aim and specific questions asked, especially for those assessing acceptability of an intervention or process evaluations. Furthermore, many stakeholders (e.g. systems-level and key decision-makers) are under-represented/evaluated, and participants are likely to be people who are more motivated to respond. The sample size in many studies is relatively small. Limited studies assessed researchers, policy makers, industry representatives, government or public health views. The views of patients are limited mostly to acceptability of specific screening tests/technology; and further consultation of patient/public groups is necessary in the development of any screening programme.^11^

## Conclusions

Generally, most stakeholders are positive about screening, but strong evidence and guideline recommendation will be required for successful implementation. There are many systemic issues that will need to be addressed for successful implementation at scale, but methodologies now available seem to be accepted by many stakeholders as a practical mechanism for widespread adoption if barriers can be addressed. Given the central importance of system-level barriers, more research is needed on the perspectives and needs of system-level stakeholders, key decision-makers, and consumer groups. Additionally, further research is required to identify strategies for how to address barriers in specific healthcare jurisdictions. It is important to identify clear leadership, establish collaboration, and address systemic issues including remuneration/reimbursement and screening pathways. This will be particularly important if large RCTs and meta-analyses confirm efficacy for stroke prevention.

## Supplementary Material

euag051_Supplementary_Data

## Data Availability

All data relevant to the study are included in the article or uploaded as Supplementary information.
