# Supplementary material for: Key stakeholder views on atrial fibrillation screening: a systematic mixed-studies review and interpretive analysis
Source: Europace. 2026 Mar 14;28(4):euag051. doi: 10.1093/europace/euag051 (PMC13034532; doi:10.1093/europace/euag051)
Supplement: euag051_Supplementary_Data [file euag051_supplementary_data.zip › Supplement 1_Search Strategy.pdf]

Appendix – Search Strategies

Strategies all run 9th February 2023

Medline via Ovid

Ovid MEDLINE(R) and Epub Ahead of Print, In-Process, In-Data-Review & Other Non-Indexed Citations, Daily and Versions <1946 to February 08, 2023>

1 exp Atrial Fibrillation/ or (auricular fibrillation* or atrial fibrillation* or atrial flutter* or "a fib" or AF).ti,ab,kw,kf. 121717

2 (screen* or detect* or identif* or "case finding" or self-monitor*).ti,ab,kw,kf. or exp Mass Screening/ or (early adj3 diagnos*).ti,ab,kw,kf. or exp Early Diagnosis/ 6720127

3 exp Stakeholder Participation/ or stakeholder*.ti,ab,kw,kf. or exp Patient Satisfaction/ or (satisf* or opinion* or feasib*).ti,ab,kw,kf. or (barrier* or obstacle* or facilitat* or enabl*).ti,ab,kw,kf. or exp Qualitative Research/ or qualitative.ti,ab,kw,kf. or exp Process Assessment, Health Care/ or exp Program Evaluation/ or ((process* or program*) adj3 (assess* or evaluat*)).ti,ab,kw,kf. or exp Focus Groups/ or focus group*.ti,ab,kw,kf. or attitude*.ti,ab,kw,kf. or exp "Attitude of Health Personnel"/ or exp Health Knowledge, Attitudes, Practice/ or exp Attitude to Health/ or observ*.ti,ab,kw,kf. or exp Observation/ or exp Observational Study/ or exp Interview/ or interview*.ti,ab,kw,kf. or (survey* or questionnaire*).ti,ab,kw,kf. or exp "Surveys and Questionnaires"/ or Process Assessment, Health Care/ or mixed method*.ti,ab,kw,kf. 8351827

4 1 and 2 and 3 10565

5 limit 4 to (english language and yr="2005 -Current") 8986

Embase via Ovid

Embase <1974 to 2023 February 08>

1 exp *heart atrium fibrillation/ or exp *atrial fibrillation/ or (auricular fibrillation* or atrial fibrillation* or atrial flutter* or "a fib" or AF).ti,ab. 191793

2 (screen* or detect* or identif* or "case finding" or self-monitor* or (early adj3 diagnos*)).ti,ab,kw,kf. or exp *Mass Screening/ or *Early Diagnosis/ 8820782

3 (stakeholder* or satisf* or opinion* or feasib* or barrier* or obstacle* or facilitat* or enable* or qualitative* or ((process* or program*) adj3 (assess* or evaluat*)) or " focus group*" or attitude* or observ* or interview* or survey* or questionnaire* or "mixed method*").ti,ab. or *Stakeholder encouragement/ or *Patient Satisfaction/ or exp *Qualitative Research/ or exp *Program Evaluation/ or exp *attitude/ or *Observation/ or *Observational Study/ or exp *health care survey/ or exp *health survey/ 9708322

4	1 and 2 and 3	18029
5	limit 4 to (english language and embase and yr="2005 -Current")	6013

PsycInfo via Ebsco

#	Query	Limiters/Expanders	Last Run Via	Results
S1	TI("auricular fibrillation*" or "atrial fibrillation*" or "atrial flutter*" or "a fib" or AF) or AB("auricular fibrillation*" or "atrial fibrillation*" or "atrial flutter*" or "a fib" or AF)	Expanders - Apply equivalent subjects Search modes - Boolean/Phrase	Interface - EBSCOhost Research Databases Search Screen - Basic Search Database - APA PsycInfo	2,955
S2	DE "Heart Fibrillation"	Expanders - Apply equivalent subjects Search modes - Boolean/Phrase	Interface - EBSCOhost Research Databases Search Screen - Basic Search Database - APA PsycInfo	779
S3	S1 OR S2	Expanders - Apply equivalent subjects Search modes - Boolean/Phrase	Interface - EBSCOhost Research Databases Search Screen - Basic Search Database - APA PsycInfo	3,106
S4	TI(screen* or detect* or identif* or "case finding" or self-monitor* or (early n3 diagnos*)) or AB(screen* or detect* or identif* or "case finding" or self-monitor* or (early n3 diagnos*))	Expanders - Apply equivalent subjects Search modes - Boolean/Phrase	Interface - EBSCOhost Research Databases Search	897,257

			Screen - Basic Search Database - APA PsycInfo	
S5	DE "Screening" OR DE "Screening Tests"	Expanders - Apply equivalent subjects Search modes - Boolean/Phrase	Interface - EBSCOhost Research Databases Search Screen - Basic Search Database - APA PsycInfo	18,564
S6	S4 OR S5	Expanders - Apply equivalent subjects Search modes - Boolean/Phrase	Interface - EBSCOhost Research Databases Search Screen - Basic Search Database - APA PsycInfo	898,325
S7	TI(stakeholder* or satisf* or opinion* or feasib* or barrier* or obstacle* or facilitat* or enable* or qualitative* or ((process* or program*) n3 (assess* or evaluat*)) or " focus group*" or attitude* or observ* or interview* or survey* or questionnaire* or "mixed method*") or AB(stakeholder* or satisf* or opinion* or feasib* or barrier* or obstacle* or facilitat* or enable* or qualitative* or ((process* or program*) n3 (assess* or evaluat*)) or " focus group*" or attitude* or observ* or interview* or survey* or questionnaire* or "mixed method*")	Expanders - Apply equivalent subjects Search modes - Boolean/Phrase	Interface - EBSCOhost Research Databases Search Screen - Basic Search Database - APA PsycInfo	1,942,591
S8	(((((((((DE "Stakeholder") OR (DE "Client Satisfaction")) AND (DE "Qualitative Measures" OR DE "Qualitative Methods" OR DE "Focus Group" OR DE "Grounded Theory" OR DE "Interpretative Phenomenological Analysis" OR DE "Narrative Analysis" OR DE "Semi-Structured Interview" OR DE "Thematic Analysis")) AND (DE "Focus Group" OR DE "Focus Group Interview" OR DE "Focus Group Interview")) AND (DE "Attitude Change" OR DE "Attitude Formation" OR DE "Frame of	Expanders - Apply equivalent subjects Search modes - Boolean/Phrase	Interface - EBSCOhost Research Databases Search Screen - Basic Search	2,480

	Reference" OR DE "Attitudes" OR DE "Abortion (Attitudes Toward)" OR DE "Adolescent Attitudes" OR DE "Adult Attitudes" OR DE "Aged (Attitudes Toward)" OR DE "Aging (Attitudes Toward)" OR DE "Attitude Change" OR DE "Attitude Formation" OR DE "Attitude Similarity" OR DE "Child Attitudes" OR DE "Childrearing Attitudes" OR DE "Client Attitudes" OR DE "Community Attitudes" OR DE "Computer Attitudes" OR DE "Consumer Attitudes" OR DE "Counselor Attitudes" OR DE "Cultural Attitudes" OR DE "Death Attitudes" OR DE "Disabled (Attitudes Toward)" OR DE "Drug Usage Attitudes" OR DE "Eating Attitudes" OR DE "Educational Employee Attitudes" OR DE "Employee Attitudes" OR DE "Employer Attitudes" OR DE "Environmental Attitudes" OR DE "Explicit Attitudes" OR DE "Family Planning Attitudes" OR DE "Female Attitudes" OR DE "Gender Role Attitudes" OR DE "Health Attitudes" OR DE "Health Personnel Attitudes" OR DE "Ideology" OR DE "Implicit Attitudes" OR DE "Job Applicant Attitudes" OR DE "Law Enforcement Employee Attitudes" OR DE "Male Attitudes" OR DE "Marriage Attitudes" OR DE "Obesity (Attitudes Toward)" OR DE "Occupational Attitudes" OR DE "Parental Attitudes" OR DE "Paternalism" OR DE "Political Attitudes" OR DE "Preferences" OR DE "Psychologist Attitudes" OR DE "Public Opinion" OR DE "Racial and Ethnic Attitudes" OR DE "Sex Role Attitudes" OR DE "Sexual Attitudes" OR DE "Socioeconomic Class Attitudes" OR DE "Sports (Attitudes Toward)" OR DE "Stereotyped Attitudes" OR DE "Student Attitudes" OR DE "Teacher Attitudes" OR DE "Work (Attitudes Toward)" OR DE "World View")) OR (DE "Attitude Measures" OR DE "Semantic Differential")) OR (DE "Observation Methods" OR DE "Direct Observation" OR DE "Participant Observation")) OR (DE "Surveys" OR DE "Consumer Surveys" OR DE "Mail Surveys" OR DE "Online Surveys" OR DE "Telephone Surveys")) OR (DE "Questionnaires" OR DE "General Health Questionnaire")) AND (DE "Interviewing" OR DE "Interviewers" OR DE "Interviews" OR DE "Cognitive Interview" OR DE "Focus Group Interview" OR DE "Intake Interview" OR DE "Interview Schedules" OR DE "Job Applicant Interviews" OR DE "Psychodiagnostic Interview" OR DE "Semi-Structured Interview")) OR (DE "Mixed Methods Research")		Database - APA PsycInfo	
S9	S7 OR S8	Expanders - Apply equivalent subjects Search modes - Boolean/Phrase	Interface - EBSCOhost Research Databases Search Screen - Basic Search Database - APA PsycInfo	1,942,654

S10	S3 AND S6 AND S9	Expanders - Apply equivalent subjects<br>Search modes - Boolean/Phrase	Interface - EBSCOhost Research Databases<br>Search Screen - Basic Search<br>Database - APA PsycInfo	261
S11	S3 AND S6 AND S9	Limiters - Publication Year: 2005-2023<br>Expanders - Apply equivalent subjects<br>Search modes - Boolean/Phrase	Interface - EBSCOhost Research Databases<br>Search Screen - Basic Search<br>Database - APA PsycInfo	243
S12	S3 AND S6 AND S9	Limiters - Publication Year: 2005-2023<br>Expanders - Apply equivalent subjects<br>Narrow by Language: - english<br>Search modes - Boolean/Phrase	Interface - EBSCOhost Research Databases<br>Search Screen - Basic Search<br>Database - APA PsycInfo	221

CINAHL via Ebsco

#	Query	Limiters/Expanders	Last Run Via	Results
S1	TI("auricular fibrillation*" or "atrial fibrillation*" or "atrial flutter*" or "a fib" or AF) or AB("auricular fibrillation*" or "atrial fibrillation*" or "atrial flutter*" or "a fib" or AF)	Expanders - Apply equivalent subjects<br>Search modes - Boolean/Phrase	Interface - EBSCOhost Research Databases<br>Search Screen - Advanced Search	39,513

			Database - CINAHL	
S2	(MH "Atrial Fibrillation")	Expanders - Apply equivalent subjects Search modes - Boolean/Phrase	Interface - EBSCOhost Research Databases Search Screen - Advanced Search Database - CINAHL	29,475
S3	S1 OR S2	Expanders - Apply equivalent subjects Search modes - Boolean/Phrase	Interface - EBSCOhost Research Databases Search Screen - Advanced Search Database - CINAHL	45,536
S4	TI(screen* or detect* or identif* or "case finding" or self-monitor* or (early n3 diagnos*)) or AB(screen* or detect* or identif* or "case finding" or self-monitor* or (early n3 diagnos*))	Expanders - Apply equivalent subjects Search modes - Boolean/Phrase	Interface - EBSCOhost Research Databases Search Screen - Advanced Search Database - CINAHL	1,189,580
S5	(MH "Health Screening+") OR (MH "Early Diagnosis+")	Expanders - Apply equivalent subjects Search modes - Boolean/Phrase	Interface - EBSCOhost Research Databases Search Screen - Advanced Search Database - CINAHL	126,976

S6	S4 OR S5	Expanders - Apply equivalent subjects Search modes - Boolean/Phrase	Interface - EBSCOhost Research Databases Search Screen - Advanced Search Database - CINAHL	1,237,683
S7	TI(stakeholder* or satisf* or opinion* or feasib* or barrier* or obstacle* or facilitat* or enable* or qualitative* or ((process* or program*) n3 (assess* or evaluat*)) or " focus group*" or attitude* or observ* or interview* or survey* or questionnaire* or "mixed method*") or AB(stakeholder* or satisf* or opinion* or feasib* or barrier* or obstacle* or facilitat* or enable* or qualitative* or ((process* or program*) n3 (assess* or evaluat*)) or " focus group*" or attitude* or observ* or interview* or survey* or questionnaire* or "mixed method*")	Expanders - Apply equivalent subjects Search modes - Boolean/Phrase	Interface - EBSCOhost Research Databases Search Screen - Advanced Search Database - CINAHL	1,658,123
S8	(MH "Stakeholder Participation") OR (MH "Patient Satisfaction+") OR (MH "Qualitative Studies+") OR (MH "Multimethod Studies") OR (MH "Process Assessment (Health Care)+") OR (MH "Program Evaluation") OR (MH "Focus Groups") OR (MH "Attitude+") OR (MH "Attitude of Health Personnel+") OR (MH "Attitude to Illness+") OR (MH "Observational Methods+") OR (MH "Surveys+") OR (MH "Questionnaires+") OR (MH "Interviews+")	Expanders - Apply equivalent subjects Search modes - Boolean/Phrase	Interface - EBSCOhost Research Databases Search Screen - Advanced Search Database - CINAHL	1,249,499
S9	S7 OR S8	Expanders - Apply equivalent subjects Search modes - Boolean/Phrase	Interface - EBSCOhost Research Databases Search Screen - Advanced Search	2,207,346

			Database - CINAHL	
S10	S3 AND S6 AND S9	Expanders - Apply equivalent subjects<br>Search modes - Boolean/Phrase	Interface - EBSCOhost Research Databases<br>Search Screen - Advanced Search<br>Database - CINAHL	3,277
S11	S3 AND S6 AND S9	Limiters - Published Date: 20050101-20231231<br>Expanders - Apply equivalent subjects<br>Search modes - Boolean/Phrase	Interface - EBSCOhost Research Databases<br>Search Screen - Advanced Search<br>Database - CINAHL	3,022
S12	S3 AND S6 AND S9	Limiters - Published Date: 20050101-20231231<br>Expanders - Apply equivalent subjects<br>Narrow by Language: - english<br>Search modes - Boolean/Phrase	Interface - EBSCOhost Research Databases<br>Search Screen - Advanced Search<br>Database - CINAHL	2,971

Scopus

TITLE-ABS ("auricular fibrillation*" OR "atrial fibrillation*" OR "atrial flutter*" OR "a fib" OR "AF") AND TITLE-ABS (screen* OR detect* OR identif* OR "case finding" OR self-monitor* OR (early W/3 diagnos*)) AND TITLE-ABS (stakeholder* OR satisf* OR opinion* OR feasib* OR barrier* OR obstacle* OR facilitat* OR enable* OR qualitative* OR ((process* OR program*) W/3 (assess* OR evaluat*)) OR " focus group*" OR attitude* OR observ* OR interview* OR survey* OR questionnaire* OR "mixed method*") AND (LIMIT-TO (PUBYEAR , 2023) OR LIMIT-TO (PUBYEAR , 2022) OR LIMIT-TO (PUBYEAR , 2021) OR LIMIT-TO (PUBYEAR , 2020) OR LIMIT-TO (PUBYEAR , 2019) OR LIMIT-TO (PUBYEAR , 2018) OR LIMIT-TO (PUBYEAR , 2017) OR LIMIT-TO (PUBYEAR , 2016) OR LIMIT-TO (PUBYEAR , 2015) OR LIMIT-TO (PUBYEAR , 2014) OR LIMIT-TO (PUBYEAR , 2013) OR LIMIT-TO (PUBYEAR , 2012) OR LIMIT-TO (PUBYEAR , 2011) OR LIMIT-TO (PUBYEAR , 2010) OR LIMIT-TO (PUBYEAR , 2009) OR LIMIT-TO (PUBYEAR , 2008) OR LIMIT-TO (PUBYEAR , 2007) OR LIMIT-TO (PUBYEAR , 2006) OR LIMIT-TO (PUBYEAR , 2005)) AND (LIMIT-TO (LANGUAGE , "English")) View less

Web of Science Core Collection

Web of Science Search Strategy (v0.1)

Database: Web of Science Core Collection

Entitlements:

- WOS.IC: 1993 to 2023
- WOS.CCR: 1985 to 2023
- WOS.SCI: 1900 to 2023
- WOS.AHCI: 1975 to 2023
- WOS.BHCI: 2008 to 2023
- WOS.BSCI: 2008 to 2023
- WOS.ESCI: 2018 to 2023
- WOS.ISTP: 1990 to 2023

- WOS.SSCI: 1956 to 2023

- WOS.ISSHP: 1990 to 2023

Searches:

1: TS= ("auricular fibrillation*" or "atrial fibrillation*" or "atrial flutter*" or "a fib" or AF) Date Run: Thu Feb 09 2023 10:25:18 GMT+0000 (Greenwich Mean Time) Results: 161243

2: TS=(screen* or detect* or identif* or "case finding" or self-monitor* or (early near/3 diagnos*)) Date Run: Thu Feb 09 2023 10:25:24 GMT+0000 (Greenwich Mean Time) Results: 10508705

3: TS=(stakeholder* or satisf* or opinion* or feasib* or barrier* or obstacle* or facilitat* or enable* or qualitative* or ((process* or program*) near/3 (assess* or evaluat*)) or "focus group*" or attitude* or observ* or interview* or survey* or questionnaire* or "mixed method*") Date Run: Thu Feb 09 2023 10:25:35 GMT+0000 (Greenwich Mean Time) Results: 13977923

4: #1 AND #2 AND #3 Timespan: 2005-01-01 to 2023-12-31 Date Run: Thu Feb 09 2023 10:26:36 GMT+0000 (Greenwich Mean Time) Results: 8971

5: #1 AND #2 AND #3 and English (Languages) Timespan: 2005-01-01 to 2023-12-31 Date Run: Thu Feb 09 2023 10:26:43 GMT+0000 (Greenwich Mean Time) Results: 8769
